# A case of Raine syndrome presenting with facial dysmorphy and review of literature

**DOI:** 10.1186/s12881-018-0593-x

**Published:** 2018-05-11

**Authors:** Jayesh Sheth, Riddhi Bhavsar, Ajit Gandhi, Frenny Sheth, Dhairya Pancholi

**Affiliations:** 1 FRIGE’s Institute of Human Genetics, FRIGE House, Jodhpur Gam Road, Satellite, Ahmedabad, 380015 India; 2 Unique Hospital, Main Road, South Kasba, Solapur, 413007 India

**Keywords:** Case report, Developmental delay, Facial dysmorphy, *FAM20C* gene, Osteosclerosis, Raine syndrome

## Abstract

**Background:**

Raine syndrome (RS) – an extremely rare autosomal recessive genetic disorder, is caused by a biallelic mutation in the *FAM20C* gene. Some of the most common clinical features include generalized osteosclerosis with a periosteal bone formation, dysmorphic face, and thoracic hypoplasia. Many cases have also been reported with oro-dental abnormalities, and developmental delay. Most of the cases result in neonatal death. However, a few non-lethal RS cases have been reported where patients survive till adulthood and exhibits a heterogeneous clinical phenotype. Clinical diagnosis of RS has been done through facial appearance and radiological findings, while confirmatory diagnosis has been conducted through a molecular study of the *FAM20C* gene.

**Case presentation:**

A 6-year-old girl was born to healthy third degree consanguineous parents. She presented with facial dysmorphy, delayed speech, and delayed cognition. Radiography showed small sclerotic areas in the lower part of the right femur, and an abnormally-shaped skull with minimal sclerosis in the lower occipital region. Computer tomography scan of the brain revealed mild cortical atrophy, and MRI scan of the brain showed corpus callosal dysgenesis with the absence of the rostral area. Chromosome banding at 500 band resolution showed a normal female karyotype. No quantitative genomic imbalance was detected by aCGH. Further study conducted using Clinical Exome Sequencing identified a homozygous missense variation c.1228 T > A (p.Ser410Thr) in the exon 6 of *FAM20C* gene – a likely pathogenic variant that confirmed the clinical diagnosis of RS. The variant was confirmed in the proband and her parents using Sanger sequencing. Prenatal diagnosis during subsequent pregnancy revealed heterozygous status of the fetus, and a normal carrier child was delivered at term.

**Conclusions:**

The syndrome revealed markedly variable presentations such as facial dysmorphy and developmental delay, and was localized to diffuse bone osteosclerosis. Clinical indications, striking radiological findings and molecular testing of *FAM20C* gene confirmed the diagnosis of RS. A rarity of the disorder and inconsistent phenotype hindered the establishment of genotype-phenotype correlations in RS. Therefore, reporting more cases and conducting further research would be crucial in defining the variable radiologic and molecular defects of the lethal and non-lethal forms of this syndrome.

**Electronic supplementary material:**

The online version of this article (10.1186/s12881-018-0593-x) contains supplementary material, which is available to authorized users.

## Background

Raine syndrome (OMIM #259775) is also known as osteosclerotic bone dysplasia. With the estimated prevalence of < 1 in 1,000,000, the disease is categorized as a rare autosomal recessive disorder. A mutation in the *FAM20C* (Family with sequence similarity 20, member C) gene (OMIM* 611061) located on chromosome 7p22.3 is responsible for the disease [1]. This gene encodes a member, which is a Golgi casein kinase and has an S-x-E/pS consensus motif (where S is serine, x is any amino acid and E/pS can be Glutamic acid or phosphoserine) that phosphorylates the serine residues of the extracellular proteins called several secretory calcium-binding–phosphoproteins (SCPP). The bio-mineralization of bones and teeth is carried out by a small integrin-binding ligand – N-linked glycoproteins (SIBLINGs), an SCPP due to its high affinity for calcium [2]. Also, FAM20C phosphorylates the C-terminal dentin matrix protein 1 (Dmp1) and makes it a highly negatively charged domain, which in turn recruits calcium ions and help the mineral deposition [3]. A mutation in the *FAM20C* gene is reported to cause down-regulation in Dmp1 mRNA expression and up-regulation of the fibroblast growth factor 23 (FGF23) mRNA expression, which results in FGF23-related hypophosphatemia in RS [4]. FGF23 maintains phosphate homeostasis in the body and stops reabsorption of excess phosphate in the bloodstream.

In 1989, a unique case of a neonate born with a set of clinical indications including cleft palate, hypoplastic nose, low set ears, gum hyperplasia, microcephaly, exophthalmos, and diffuse osteosclerosis of the bones was described by Raine et al. The proband expired within 3 h of her birth. This presented the first case of RS [5]. The term ‘Raine syndrome’ was proposed when 2 other cases of a rare neonatal lethal sclerotic bone disorder exhibiting similar clinical presentations were reported [6]. RS is characterized by generalized osteosclerotic bone dysplasia, periosteal bone formation, and a distinct facial phenotype. To the best of our knowledge, approximately 24 cases of RS with homozygous or compound heterozygous missense mutation, microdeletion, whole gene deletion, and splice-site mutations in the *FAM20C* gene have been reported till the year 2017 [5–28]**.**

Over time, it has been observed that RS has a broader phenotypic spectrum. Many cases of RS initially described were shown to be lethal within the first few weeks of life [5, 14, 21, 24]. However, cases have been reported where the patient lived up to teenage years or adulthood, which suggests the presence of non-lethal form of RS [8, 11, 12, 29, 30]. Heterogeneity of the clinical phenotypes ranging from mild to severe manifestation has also been observed in RS. Certain lethal cases have been described with severe osteosclerosis, brachycephaly and choanal atresia in the affected patients [9, 24]. On the other hand, despite having such anomalies, the patient survived till childhood [13]. Other clinical features like microcephaly, delayed language and fine motor skills, periapical abscesses, gingival hyperplasia and open bite malocclusion have also been observed to be associated with RS [8].

The present case reports a child exhibiting non-lethal form of RS portraying craniofacial abnormalities, osteosclerosis, and developmental delay. Upon investigation, a homozygous missense variant in the *FAM20C* gene was observed. During subsequent pregnancy, the parents and the fetus were heterozygous for the said variant.

## Case presentation

### Clinical features

A 6-year-old girl, was born to healthy parents with third-degree consanguinity. This family of Indian origin was referred for investigation and genetic counseling for facial dysmorphy with global developmental delay, and behavioral issues of the proband. Maternal history revealed intra-uterine growth retardation during the antenatal period that ended with a full-term female baby born through cesarean section due to breach presentation. Her birth weight was 2.75 kg.

**Fig. 1:**
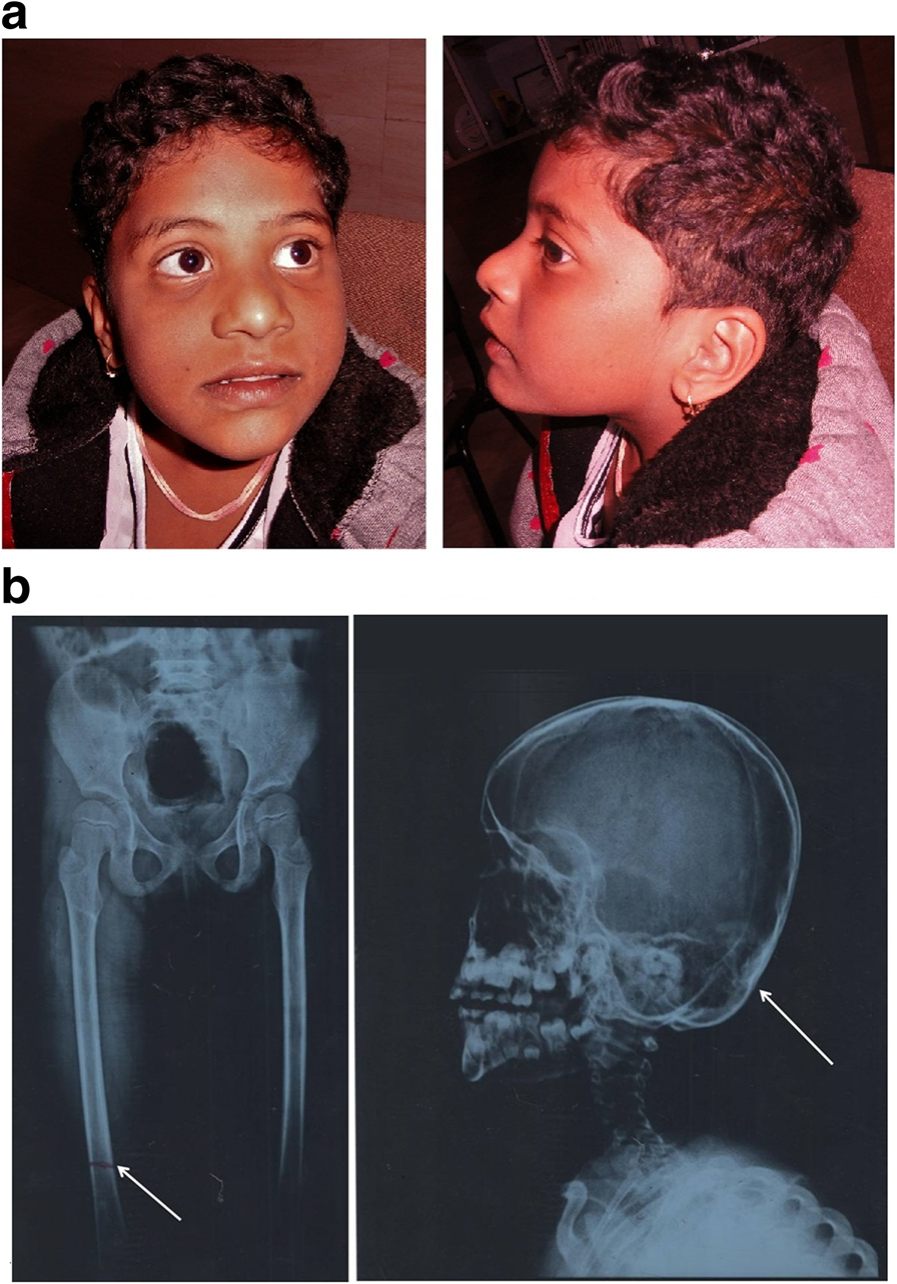
**a** Clinical picture of the proband showing features of microcephaly, narrow bifrontal diameter, flat forehead, epicanthal folds, hypertelorism, depressed and low nasal bridge with bulbous nasal tip, flaring nares, prominent philtrum and pointed chin. **b** X-ray of extremities of the proband (Left): the arrow indicates small sclerotic areas observed in lower part of the right femur; X-ray of the skull (Right): abnormal skull shape skull; the arrow indicates minimal sclerosis of lower occipital region-bones

The proband had normal feeding history, and there were no neonatal concerns. At approximately 5 months of age, the proband attained her neck control. She could stand with support by 3 years and could walk independently by 5 years of age. She could utter only a few bi-syllable words. Despite being hyperactive, no abnormal hand movements were observed. She had a history of two episodes of febrile seizures at 8 months and 2 years of age. During these episodes, she was observed with other symptoms such as cough, cold, and fever. Her physical examination revealed craniofacial disproportion, which included microcephaly with a narrow bifrontal diameter and a flat forehead. Other anomalies such as facial dysmorphy including epicanthal folds, hypertelorism, depressed and low nasal bridge with bulbous nasal tip, flaring nares, prominent philtrum, and pointed chin were also observed (Fig. 1a). However, no oro-dental anomalies were observed in the proband.

She also had hallux valgus with increased gap between the first and second toes (sandal gap deformity), clinodactyly of toes, and pes planus (flat feet). No other deformity of the trunk or limbs was observed. She had dark pigmentation in armpits, genitalia, and on the knuckles. She had mild hypotonia with brisk and deep tendon reflexes. Her expressive communication was good but her verbal communication was affected. Her social responses seemed to be fair. The proband was diagnosed with attention deficit hyperactivity disorder (ADHD) with psychomotor developmental delay. Sample collection and written informed consent was obtained according to the need of the institutional ethics committee in accordance with Helsinki declaration. CARE guidelines were followed for reporting.

### Radiological and haematological investigations

On investigation, low hemoglobin with normal serum calcium (10 mg/dl) and Vitamin B12 levels were detected. Her sleep electroencephalogram (EEG) was normal. Radiographic studies of the proband showed small sclerotic areas in the lower part of the right femur, and abnormally shaped skull with minimal sclerosis in the lower occipital region (Fig. 1b). However, the bone density was normal and there were no features of fracture or dislocation of the bones. Corticomedullary differentiation was maintained and no periosteal reaction was noted. CT scan of the brain revealed prominent sulcal pattern with enlarged cisternal spaces in the brain parenchyma. These changes were prominently observed in the frontoparietal region. In addition, mild cortical atrophy was also detected. There was no evidence of focal hypo– or hyperdense lesion. Moreover, subdural, extradural or intracerebral hemorrhage, or subarachnoid bleeding was not observed. The pons, midbrain, both cerebellar hemisphere, and brain stem appeared normal. No mass lesion was observed in the posterior fossa.

MRI revealed a significant hypoplastic appearance of posterior part of the brain. It also showed corpus callosal dysgenesis with the absence of rostral area. Moreover, a mild paucity of the peritrigonal white matter was seen surrounding the trigones of bilateral lateral ventricles. However, no obvious gliosis, extra-axial collection or lesions were noted. Her echocardiogram showed normal systemic and pulmonary venous drainage, normal valves, and normal sized cardiac chambers. The Ophthalmological evaluation revealed bilateral intact vision with healthy macula.

### Molecular genetics investigations

Chromosome banding study showed a normal female karyotype i.e. 46, XX at 550-bands resolution [20]. The fluorescence in situ hybridization (FISH) study was performed using LSI ELN (7q11) (orange) /LSI 7q22 (green) (Kreatech dual color probe). Since two signals were observed in all the cells, clinical prediction of Williams syndrome was ruled out. The proband DNA was further investigated with array comparative genomic hybridization (aCGH) using [Agilent 60 K], with an average resolution of 150 kb. No copy number variations i.e. deletions and/or duplications of pathogenic significance were detected [arr(1–22,X)× 2]. Thus, cytogenetic aberrations with resolution of 150 kb or greater were unlikely to be responsible for the clinical features in the proband.

The genomic DNA (gDNA) of the proband was isolated from the peripheral blood using the salting-out technique [31]. This DNA sample was processed for clinical exome sequencing (CES) on Illumina NextSeq 500. A detailed protocol is mentioned in the Additional file 1. Sequencing detected a homozygous missense variation c.1228 T > A in the exon 6 of the *FAM20C* gene (OMIM*611061) (GenBank accession number NM.020223.3; coding sequences NP_064608). This variation resulted in the amino acid substitution of threonine for serine at codon 410 (p.Ser410Thr; ENST00000313766), confirming the clinical diagnosis of RS (OMIM # 259775).

The functional effect of this variant was studied using the in silico analysis tools. The variant was predicted to be disease-causing by Mutation Taster (http://www.mutationtaster.org/), with a score of 58. The impact of the substitution of serine to threonine was predicted to be probably damaging (score of 0.972) by Polyphen2 (PolymorphismPhenotypingV2) (http://genetics.bwh.harvard.edu/pph2/). Scale-invariant feature transform (SIFT) (http://sift.jcvi.org/) predicted that the variant was tolerated with a score of 0.51, and PROVEAN anticipated that the variant had a deleterious effect on the protein function. This variant was reported as a likely pathogenic allele (SCV000583504.1) and benign (SCV000343602.2) in ClinVar. Its reference SNP number is rs148276213. The minor allele frequency of the rs148276213 variant is 0.0034 in the 1000 genomes database and 0.006108 in the ExAC database for the South Asian population.

CES also revealed another compound heterozygous variant in the tubulin gamma complex associated with protein 6 (*TUBGCP6)* gene (OMIM*610053) in the proband. The compound heterozygous variant in exon 24 [c.5327C > G (p.Ser1776Cys)] and exon 16 [c.3383G > A (p.Arg1128Lys)] is associated with microcephaly and chorioretinopathy-1 (OMIM#251270). In silico analysis predicted the first variant (c.5327C > G/p.Ser1776Cys) to be disease-causing using the Mutation Taster, tolerated by SIFT (score 0.07) and probably damaging by PolyPhen (score 1.00). While second variant (c.3383G > A /p.Arg1128Lys) found to be a polymorphism by Mutation T@ster, tolerated by SIFT (score 0.65), and benign by PolyPhen (score 0.023). Second variant was dismissed as the cause of disease in the proband, because this genotypic variation did not correlate with the phenotypic features of the proband, and also because various bioinformatics prediction softwares could not predict it as damaging.

**Fig. 2:**
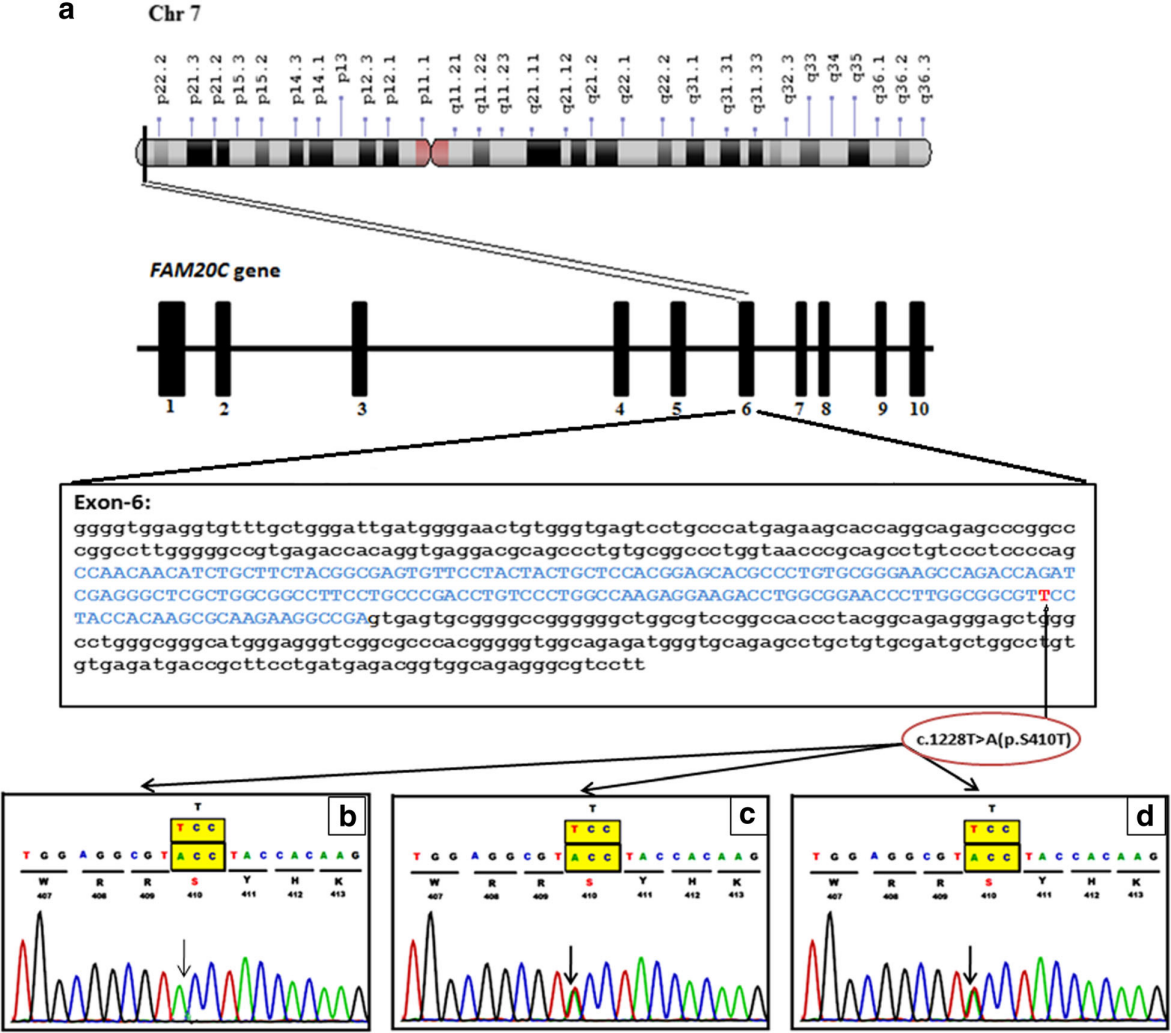
Molecular analysis. **a** Schematic representation of *FAM20C* position on chr7p22.3 and reported variants in exon 6 with c.1228 T > A /p.Ser410Thr of *FAM20C*. **b** Sequence chromatogram of the proband (Homozygous for c.1228 T > A /p.Ser410Thr in the *FAM20C* gene). **c** Sequence chromatogram of the mother (Heterozygous for c.1228 T > A/p.Ser410Thr in the *FAM20C* gene). **d** Sequence chromatogram of the father (Heterozygous for c.1228 T > A/p.Ser410Thr in the *FAM20C* gene)

The variant has an autosomal recessive mode of inheritance. Its confirmation in the proband and her parents was carried out using bi-directional Sanger sequencing with primers covering both the exon and the intron-exon boundary of exon 6 of the *FAM20C* gene (Additional file 2). Sequencing confirmed both the parents to be heterozygous, and the proband to be homozygous for c.1228 T > A (p.Ser410Thr) variant in exon 6 of *FAM20C* gene (Fig. 2a-d). This variant was submitted to ClinVar database (accession ID is SCV000583504.1). During a subsequent pregnancy, the same family approached again for genetic counseling. The prenatal diagnosis revealed a heterozygous state of the fetus for the c.1228 T > A (p.Ser410Thr) variant, and a normal carrier child was delivered (data not shown).

### Homology modeling, structure validation and protein stability due to c.1228 T > A (p.Ser410Thr) variant

Using NCBI Basic Local Alignment Search Tool (BLAST), the native and mutated sequences of the *FAM20C* gene were studied to understand the effect of the variant, and also to predict the protein structure against PDB with default parameters [32]. The template PDBID: 5WRR was considered for modeling the protein structure. Additional details regarding the homological modeling is mentioned in the Additional file 3 [33–41].

**Fig. 3:**
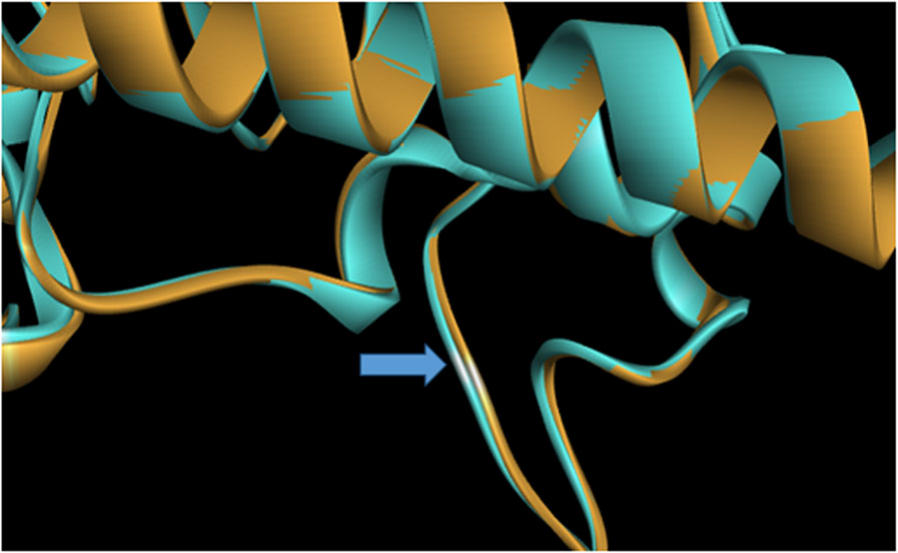
Homology modeling, structure validation and protein stability due to c.1228 T > A (p.Ser410Thr) variant: The mutation has occurred in exon 6 of *FAM20C* gene at codon number 410 causing codon change from TCC to ACC. The super imposed model of native structure (blue) and mutant structure (brown) produced using Discovery Studio software for mutation p.Ser410Thr shows conformational changes in the loop regions; indicated by blue arrow

A root mean sequence deviation (RMSD) of 0.3 A^o^ was observed between native and mutant structure, indicating changes in the loop regions of the superimposed structure. A decrease instability was predicted by iStable with a confidence score of 0.605. Furthermore, I-Mutant predicted a large decrease in protein stability due to the variant p.Ser410Thr with ΔΔG value of − 1.37 Kcal/mol (Fig. 3).

### Conservation of the FAM20C p.Ser410Thr residue in orthologs

**Fig. 4:**
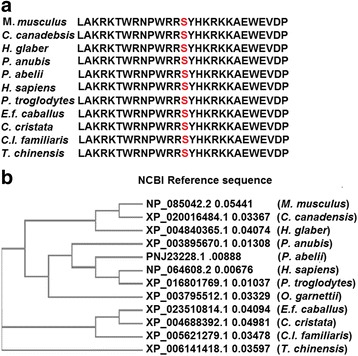
Conservation of *FAM20C* (p.Ser410Thr) residue in various orthologs: (**a**) The multiple alignment of the protein sequence region surrounding the variant c.1228 T > A (p.Ser410Thr) against various orthologous sequences. The conserved Serine (S) residue in all the orthologs is mark red. **b** The phylogenetic tree depicting the evolutionary conservation of *FAM20C* gene in various orthologs

The protein sequence of *Homo sapiens* (NP_064608) was aligned along with other species using an online multiple sequence alignment program known as Clastal Omega (https://www.ebi.ac.uk/Tools/msa/clustalo/). It is observed that the orthologs protein sequences of the *FAM20C* gene were highly identical to *H. sapiens FAM20C* gene protein sequence suggesting a highly conserved locus (Fig. 4a, b; Additional file 4).

### Population screening for c.1228T>A (p.Ser410Thr) variant by ARMS-PCR

Using PCR technique of amplification-refractory mutation system (ARMS), 200 unrelated control samples (100 males and 100 females) were studied to demonstrate the pathogenicity of c.1228T>A (p.Ser410Thr) variant. Amplification of the PCR product was confirmed by 2.5% agarose gel electrophoresis. From the 200 subjects screened for the above genotype, no carriers for the c.1228T>A (p.Ser410Thr) variant were found (Additional file 5).

## Discussion and conclusions

Most of the RS cases initially reported died at an infantile age. The prevalence of non-lethal RS has been reported in few cases over time. The most striking features of RS are osteosclerosis and facial anomalies [42]. The case presented here exhibited facial dysmorphism such as a flat forehead, epicanthal folds, hypertelorism, depressed and low nasal bridge with bulbous nasal tip, flaring nares, prominent philtrum, and pointed chin. Such phenotypical appearances have also been observed in other lethal and non-lethal RS cases [7, 8, 20]. These developmental delays was reported in two siblings affected with RS from two different families [8, 28]. Developmental delay is a very rare phenomenon observed amongst RS patients. Additionally, bone abnormalities like osteosclerosis, hallux valgus, sandal gap deformity**,** clinodactyly of toes, and pes planus were observed. Several cases of RS reported so far, have shown a broad range of bone anomalies including thoracic hypoplasia, metaphyseal flaring and under-mineralized distal phalanges [14, 43]. Thus, it can be concluded that there is an association of RS with heterogeneous clinical phenotypes.

Several mutations in the *FAM20C* gene have consistently been reported in RS patients (Table 1). In our case, homozygous missense variant in exon 6 of *FAM20C* gene was identified. The identified variant was also reported as benign by Emory University. Mutations in orthologs of the *FAM20C* gene have also exhibited a similar effect in different species. A group of Border Collies represented the canine model for human Raine syndrome. A recessive transmission of c.899C>T (p.A300V) mutation in *FAM20C* gene resulted in mineralization defect in these canines [44]. In a murine model, specific ablation of the *FAM20C* gene in cells expressing type I collagen led to skeletal defects and hypophosphatemia [45]. This suggests that *FAM20C* gene function is highly conserved amongst orthologs.

**Table 1:** Genotype and Phenotypic Variability in Some Reported Cases of Raine syndrome

Sr No.	Mutation(s) in *FAM20C* gene	Exon/ intron of mutation	Pattern of mutation	Sex	consanguinity	Fatality	Origin (Ethnicity)	Clinical Indications of the patients				Reference [Reference number in text]
								Bone anomalies	facial dysmorphic appearance	orodental abnormalities	Developmental delay	
1	c.1603C >T (p.Arg535Trp)	Exon 10	Homozygous missense mutation	M	Yes	neonatal death	Caucasian	Periosteal bone formation, Osteosclerosis, Thoracic Hypoplasia, brachycephaly with very large anterior and posterior fontanelles, prenatal fractures of both clavicles and some ribs	Prominent forehead, short neck, proptosis, midfacial hypoplasia, depressed nasal bridge, exophthalmos, hypoplastic nose with choanal atresia, high arched palate with mid-line groove but no cleft, hyperplastic nodular gums, low set ears, small jaw	Not Reported	Not Reported	Kingston et al., 1991 [9] Simpson et al., 2007 [21]
2	c.915-3C>G	Intron 4/Exon 5 acceptor splice-site change	Homozygous splice site mutation	M	No	neonatal death	Arab	Periosteal bone formation, Osteosclerosis, Thoracic Hypoplasia, and marked bowing of the femurs, tibiae, ulnae, short limbs	Prominent forehead, short neck, proptosis, midfacial hypoplasia, depressed nasal bridge, wide anterior fontanelle, exophthalmos, bilateral choanal atresia, large protruding tongue	Not Reported	Not Reported	Al-Gazali et al., 2003 [24] Simpson et al., 2007 [21]
3	c.1121T>G (p.Leu374Arg)	Exon 6	Homozygous missense mutation	F	Yes	neonatal death	Turkish	Periosteal bone formation, Osteosclerosis, Thoracic Hypoplasia, Pulmonary Hypoplasia, hypoplastic fingernails, Brachydactyly, metaphyseal flaring, narrow medula	Bi-temporally narrowed forehead, short neck, proptosis, midfacial hypoplasia, depressed nasal bridge, carp-shaped mouth with narrow lips, large low set ears, posteriorly rotated ears, smooth and prominent philtrum	Gingival hyperplasia	Diffused intracerebral Calcifications, microcephaly	Hulskamp et al., 2003 [14] Simpson et al., 2007 [21]
4	45, XY psudic (7;7) (p22;p22)	NA	Chromosome 7 rearrangement and micro deletion. *FAM20C* gene is located within the deleted region	M	No	neonatal death (died 2 h after birth)	Unknown	Lethal osteosclerotic bone dysplasia, irregular periosteal bone formation along the clavicles and ribs, skull had wide cranial sutures with evidence of premature closure, base of the skull showed an increased thickness of all bony landmarks, pulmonary Hypoplasia, Thoracic Hypoplasia	Prominent forehead, short neck, proptosis, midfacial hypoplasia, and a depressed nasal bridge	Not Reported	Not Reported	Simpson et al., 2007 [21]
5	c.1093G>A (p.Gly365Arg)	Exon 6	Homozygous	M	Yes	neonatal death	Unknown	Periosteal bone formation, Osteosclerosis, Thoracic Hypoplasia, Pulmonary Hypoplasia	Prominent forehead, short neck, proptosis, midfacial hypoplasia, depressed nasal bridge	Not Reported	Not Reported	Simpson et al., 2007 [21]
6	c.1094G>A (p.Gly365Glu)	Exon 6	Compound heterozygous mutation	F	No	neonatal death	Unknown	Periosteal bone formation, Osteosclerosis	Prominent forehead, short neck, proptosis, midfacial hypoplasia, depressed nasal bridge	Unknown	Cerebral Calcifications	Simpson et al., 2007 [21]
7	c.1322-2A>G	Intron 7 /Exon 8	Intron 7/Exon 8 acceptor splice-site mutation									
8	c.914+5G>C	Exon 4 /Intron 4	Exon 4/Intron 4 donor splice site mutation	F	No	neonatal death	Unknown	Periosteal bone formation, Osteosclerosis, Thoracic Hypoplasia, Pulmonary Hypoplasia	Prominent forehead, short neck, proptosis, midfacial hypoplasia, depressed nasal bridge	Unknown	Cerebral Calcifications	Simpson et al., 2007 [21]
9	c.1404-1G>A	Intron 8 /Exon 9	Intron 8/Exon 9 acceptor splice site mutation									
10	c.1309G>A (p.Asp437Asn)	Exon 7	Homozygous missense mutation	M	Yes	(Age 8 years at the time of inverstigation) The details of death is unavailable	Unknown	Sclerosing bone dysplasia, metaphyseal sclerosis of the long bones, diffuse abnormalities of the skull, thickening and coarse trabeculation, prominent mastoid bulges, short stature	Brachycephaly, downslanted eyes, hypoplastic nose, small downturned mouth, proptosis, turribrachycephaly, plagiocephaly, downslanting palpebral fissures, proptosis, depressed nasal bridge, small nose, protruding tongue, thick alveolar margins, low-set ears	High palate, abnormal teeth	Hydrocephalus, impaired early development, with an increase in age severe developmental delay observed	Simpson et al., 2009 [11]
11	c.796G>A (p.Gly266Arg)	Exon 2	Compound Heterozygous missense mutation	M	No	(Age 11 years at the time of inverstigation). The details of death is unavailable	Unknown	Sclerosing bone dysplasia, pectus excavatum, bulbous fingertips, thick fingers, large halluces, short stature	Turribrachycephaly, hypertelorism, arched eyebrows, an inferiorly placed right eye, low-set and protuberant ears, flat nasal bridge with rounded and bulbous nasal tip and prominent alae nasi, sunken midface, wide mouth with large tongue, relative prognathism	Secondarily edentulous	Hydrocephalus, impaired early development, with an increase in age severe developmental delay observed	Simpson et al., 2009 [11]
12	c.796G>A (p.Gly266Arg)	Exon 3										
13	c.1630C>T (p.Arg544Trp)	Exon 10	Homozygous missense mutation	M	Yes	died 38 days after birth	Indian subcontinent	Wide metaphysic, cortical hyperostosis, Osteosclerosis, bowed femur, narrow thorax	Cleft palate, long philtrum, open mouth appearance, midfacial hypoplasia, flat face, depressed nasal bridge, small nose, choanal atresia, entropion of eyelids, proptosis, low set ears, prominent forehead, clover leaf skull	Hypoplastic maxilla (excluding the molar region), small mandible/ micrognathia,	Microcephaly	Kochar et al., 2010 [20]
14	c.940C>T (p.Pro314Ser) (two siblings; Case 1 and Case 2)	Exon 4	Homozygous missense mutation	F (*n* = 2)	yes	(Age 1 year and 4 years at the time of investigation) The details of death is unavailable	Algerian	Case 1: Osteosclerosis Case 2: cerebral calcifications within parieto-occipital and periventricular white matter, increased density of vertebral bodies and calcifications of several intervertebral disks, presence of chain-like calcifications	Case 1: High forehead, hypertelorism with bilateral epicanthal folds and slightly downslanting palpebral fissures, nasal root hypoplasia, anteverted nares, dysplastic and posteriorly angulated ears with prominent lobule. Case 2: brachycephaly, bilateral epicanthal folds, midface and nasal root hypoplasia with absence of nasal crest and micrognathia.	High palate, small teeth with enamel dysplasia.	Normal psychomotor development at the time of investigation	Fradin et al., 2011 [13]
15	c.803C>T (p.Thr268Met)	Exon 3	Compound heterozygous missense mutation	M (*n* = 2)	No	(Age 18 years at the time of investigation). The details of death is unavailable	Norway	Osteosclerosis, short distal phalanges	Dolicocephaly, a narrow face with a narrow malar region, prominent forehead, depressed nasal bridge, low set eyes, hypotelorism, prognathism, a high arched palate with a midline ridge, small mouth, flat malar area	Tooth decay (evident by approximately 18 months of age) periapical abscesses. enlarged pulp chambers, elongated pulp horns up to the enamel-dentin junction, globular defects in the dentine, gingival hyperplasia	Slightly delayed language and fine motor skills at age 11 years.	Rafaelsen et al., 2013 [28]
16	c.915C>A (p.Y305X)	Exon 4				Age 16 years at the time of investigation). The details of death is unavailable						
17	46,XY.arr[hg19] 7p22.3 (36480-523731)	chromosome 7p22.3	Homozygous Complex rearrangement of chromosome and deletion of 487-kb at chromosomal location 7p22.3 that contains *FAM20C* gene	Unknown	Unknown	The details of death is unavailable	Unknown	Diffuse osteosclerosis, appositional new bone formation, the obliteration of the medullary cavities, narrow thorax, pseudo-rib fractures, small distal phalanges	Prominent forehead, eye proptosis, severe depression of the nasal bridge with short upturned nose, midface hypoplasia, micrognathia, protruding tongue,	Unknown	Unknown	Ababneh et al., 2013 [43]
18	c.1222C>T (p.Arg408Trp)	Exon 6	Homozygous mutation	M	Yes	(Age 61years at the time of investigation). The details of death is unavailable	Japanese	Hypophosphatemic osteomalacia, periosteal bone formation in the long bones, bone mineral density in the femoral neck, ossification of the posterior longitudinal ligament	Not reported	Worn out teeth, dental demineralization, loss of all teeth by the age of 17 years	Not reported	Takeyari et al., 2014 [29]
19	c.784+ 5G>C* (p.Trp202Cysfs*37) (three siblings)	After Exon 2	Homozygous donor splice site mutation	F (*n* = 1) M (*n* = 2)	Yes	(Age 21 years; 22 years and 27 years at the time of investigation). The details of death is unavailable	Brazilian	Case 2: Short fingers	Case 1,2,3: Dysplastic ears, midface hypoplasia, exophthalmos,	Case 1,2,3: Dental caries, calculus, severe gingivitis, dental plaque, open bite malocclusion, abnormal enamel, high arched and narrow palate, micrognathia, periapical abscesses, yellow brownish discoloration of teeth	Case 1: Intracranial calcifications, Case 2: microcephaly, Case 3: microcephaly,	Acevedo et al., 2015 [8]
20	c.1487C>T (p.Pro496Leu) (two siblings)	Exon 9	Homozygous missense mutation	M (*n* = 2)	Yes	(Age 13 years and 12 years at the time of investigation). The details of death is unavailable	Brazilian	Case1,2: small hands with bulbous fingertips and clinodactyly of the fifth fingers, under-mineralized distal phalanges, shaft and growth plate under-mineralization, bowing of the radius bones	Case 1,2: choanal atresia, low set ears, a hypoplastic nose with depressed nasal bridge, prominent alae nasae, down-slanting palpebral fissures and exophthalmos	Case 1,2: midface hypoplasia, micrognathia, high arched and narrow palate, enlarged gingival and palatal mucosa, unerupted permanent teeth, hypoplastic AI. recurrent periapical dental abscesses	Case 1,2: delay in psychomotor development, microcephaly	Acevedo et al., 2015 [8]
21	c.1228T>A (p.Ser410Thr)	Exon 6	Homozygous missense mutation	unknown	unknown	The details of death is unavailable	Unknown	unknown	unknown	unknown	Unknown	Emory Genetics Laboratory; 2016 [47]
22	c.676T > A (p.Trp226Arg)	Exon 2	Homozygous missense mutation	F (*n* = 2) M (*n* = 1)	Yes	The details of death is unavailable	Moroccan	Not Reported	Not Reported	amelogenesis imperfecta	learning disability, seizures	Elalaoui et al., 2016 [30]
23	c.1135G>A (p.Gly379Arg)	Exon 6	Homozygous missense mutation	M	Yes	The details of death is unavailable	South East Asian	Osteosclerosis, widening of the metaphyses of the long bones, thick and short ribs, fracture of the mid-shaft of the left clavicle, bowing of long bones, bilateralperiventricular and parenchymal punctate calcifications	Choanal atresia, frontal bossing, proptosis, mid-face hypoplasia with a depressed nasal bridge, bulbous nasal tip, tented upper lip	Not Reported	Abnormal/dystonic movements, hypertonia, hyper reflexia	Mahmood et al., 2017 [7]
24	c.1228T>A (p.Ser410Thr)	Exon 6	Homozygous missense mutation	F	Yes	Age 6 years the time of investigation). The details of death is unavailable	Indian	Osteosclerosis, hallux valgus, sandal gap deformity, clinodactyly of toes, pes planus	Flat forehead, epicanthal folds, hypertelorism, depressed and low nasal bridge with bulbous nasal tip, flaring nares, prominent philtrum, pointed chin.	No orodental anomalies were observed.	psychomotor developmental delay	Present case

Another compound heterozygous variation in the *TUBGCP6* gene (OMIM*610053) was observed in the current proband. Mutation in this gene caused microcephaly and chorioretinopathy-1 (OMIM#251270). The prominent clinical feature for this disease was microcephaly, retinal pigmentary abnormalities, and early onset of visual impairment. The proband in the present case had microcephaly with intact vision, ruling out retinopathy. There was a lack of genotypic and phenotypic co-relation in the present variant, and the variant was not considered to be predisposing for the clinical features of the proband.

One of the most prominent clinical indications in RS are skeletal defects. Mutation/s in *FAM20C* lead to loss of function, resulting in hypophosphatemia. The tissue-nonspecific isoenzyme of alkaline phosphatase (TNSALP) substrates together with inorganic pyrophosphate gets accumulated in the extracellular niche due to mutation/s in *FAM20C* gene. This causes hindrance in the normal function of mineralization. An excess of pyrophosphate accumulation results in tooth loss, osteomalacia, and calcification of bones. Asfotase alfa, a hydroxyapatite-targeted recombinant TNSALP is being used as an enzyme replacement therapy to get rid of excess pyrophosphate [46]. However, the enzyme replacement therapy is expensive and may not be affordable to many families. In such a scenario, precise genetic counseling and prenatal diagnosis remains the preferred choice.

Though RS is synonymously read as lethal osteosclerotic bone dysplasia, several reported cases including ours show the non-lethal clinical phenotype associated with the *FAM20C* mutation. In these mild RS cases, the patient survives beyond infancy. RS reflects marked variability in presentations ranging from severe to localized, non-diffuse, and mild bone osteosclerosis. Facial appearance and striking radiological findings prompt the clinical diagnosis of RS. More number of such cases is crucial to define the variable radiologic, metabolic, and molecular defects of this seemingly lethal and non-lethal autosomal recessive syndrome.

## Additional files

Additional file 1: Molecular investigations. The file describes the method used in isolation of genomic DNA, NGS and bioinformatics tools used during analysis. (DOCX 16 kb)

Additional file 2: Sanger sequencing (Variant Confirmation Test). It describes the details of primer’s used during Sanger sequencing of the proband and parents (DOCX 15 kb)

Additional file 3: Homology modeling, structure validation and protein stability due to c.1228T>A (p.Ser410Thr) variant. File describes the influence of variant change on the protein structure (DOCX 16 kb)

Additional file 4: Conservation of the FAM20C p.Ser410Thr residue in orthologs. Conservation of the variant in orthologs and *Homo sapiens*. (DOCX 15 kb)

Additional file 5: Population screening for c.1228T>A (p.Ser410Thr) variant by ARMS-PCR. The file provides details of normal healthy individuals studied for variant (c.1228T>A (p.Ser410Thr)) by ARMS-PCR. (DOCX 15 kb)

## Abbreviations

1,25(OH)_2_D: 1,25-Dihydroxy vitamin D
aCGH: Array Comparative Genomic Hybridization
ADHD: Attention Deficit Hyperactivity Disorder
ARMS: Amplification-refractory mutation system
CES: Clinical Exome Sequencing
CNS: Central Nervous System
Dmp1: Dentin matrix protein 1
EEG: Electroencephalogram
ELN: Elastin
ExAC: The Exome Aggregation Consortium
*FAM20C*: Family with sequence similarity 20,member C
FGF23: Fibroblast growth factor 23
FISH: Fluorescence in Situ Hybridization
IUGR: Intrauterine Growth Retardation
LVEF: Left ventricular ejection fraction
NCBI: National Centre for Biotechnology Information
OMIM: Online Mendelian Inheritance in Man
PCR: Polymerase chain reaction
PDB: Protein Data Base
RMSD: root mean sequence deviation
SAVES: Structural Analysis and Verification Server
SCPP: Secretory calcium binding–phosphoproteins
SIBLINGs: Small integrin-binding ligand, N-linked glycoproteins
SIFT: Scale-invariant feature transform
TNSALP: Tissue-nonspecific isoenzyme of alkaline phosphatase
*TUBGCP6*: Tubulin gamma complex associated protein 6
YASARA: Yet Another Scientific Artificial Reality Application
