# Supplementary material for: A case of Raine syndrome presenting with facial dysmorphy and review of literature
Source: BMC Med Genet. 2018 May 11;19:76. doi: 10.1186/s12881-018-0593-x (PMC5948820; doi:10.1186/s12881-018-0593-x)
Supplement: Supplementary file 1 — Molecular investigations. The file describes the method used in isolation of genomic DNA, NGS and bioinformatics tools used during analysis. (DOCX 16 kb) [file 12881_2018_593_MOESM1_ESM.docx]

**Additional file-1**

**Molecular investigations**

**Further investigation was carried out at a molecular level. The genomic DNA (gDNA) of the proband was isolated from peripheral blood** using salting-out technique [31]. **This DNA sample was processed for Clinical Exome Sequencing (CES) using Next Generation Sequencing (NGS). In brief, g**DNA was used to perform targeted gene capture using a custom capture kit. The libraries were sequenced to mean >80-100X coverage on Illumina sequencing platform. BWA program was used for the alignment of sequences obtained for human reference genome (GRCh37/hg19). Picard was used for analyses of the obtained sequence, and GATK-Lite toolkit was used to identify variants relevant to the clinical indication. Gene annotation of the variants was performed using VEP program against the Ensembl release 75 human gene model. Clinically relevant mutations were annotated using published variants in literature and a set of diseases databases like ClinVar, OMIM, GWAS, HGMD, and SwissVar. Common variants were filtered based on allele frequency in 1000Genome, Phase 3, ExAC, EVS, dbSNP141 and 1000 Japanese Genome. Multiple algorithms such as PolyPhen-2, SIFT, Mutation Assessor, Mutation Taster and LRT were used to calculate the effect of non-synonymous variants. Clinical interpretations were based on only non-synonymous and splice site variants found in the clinical exome panel consisting of 6440 genes with more than 100X coverage. A homozygous missense variation c.1228T>A was detected in the exon 6 of *FAM20C* gene (OMIM*611061) (GenBank accession number NM.020223.3; coding sequences NP_064608) on chromosome 7:295970. This variation resulted in the amino acid substitution of Threonine for Serine at codon 410 (p.Ser410Thr; ENST00000313766), confirming the clinical diagnosis of RS (OMIM # 259775).
