# Supplementary material for: A case of Raine syndrome presenting with facial dysmorphy and review of literature
Source: BMC Med Genet. 2018 May 11;19:76. doi: 10.1186/s12881-018-0593-x (PMC5948820; doi:10.1186/s12881-018-0593-x)
Supplement: Supplementary file 2 — Sanger sequencing (Variant Confirmation Test). It describes the details of primer’s used during Sanger sequencing of the proband and parents (DOCX 15 kb) [file 12881_2018_593_MOESM2_ESM.docx]

**Additional file-2**

**Sanger sequencing (Variant Confirmation Test)**

The DNA samples of the proband and both the parents with a concentration of 250 ng/µL were amplified using Thermal Cycler-2720 (Applied Biosystems). Exon 6 of *FAM20C* gene was amplified using a forward primer sequence 5'CTTCTACGGCGAGTGTTCCT3' and reverse primer sequence 5'AGGCTCTGCACCCATCTCT3'. A total of 32 cycles of Polymerase Chain Reactions (PCR) were run with initial denaturation (94°C; 5 minutes), denaturation (94°C; 30 seconds), annealing (60°C; 45 seconds), elongation (72°C; 1 minute) and final elongation (72°C; 7 minutes). The amplifications of the PCR products were confirmed by 2.5% agarose gel electrophoresis. Using these PCR products, sequencing was carried out on the genetic analyzer ABI-3100.
