# Supplementary material for: A case of Raine syndrome presenting with facial dysmorphy and review of literature
Source: BMC Med Genet. 2018 May 11;19:76. doi: 10.1186/s12881-018-0593-x (PMC5948820; doi:10.1186/s12881-018-0593-x)
Supplement: Supplementary file 3 — Homology modeling, structure validation and protein stability due to c.1228T>A (p.Ser410Thr) variant. File describes the influence of variant change on the protein structure (DOCX 16 kb) [file 12881_2018_593_MOESM3_ESM.docx]

**Additional file-3**

**Homology modeling, structure validation and protein stability due to c.1228T>A (p.Ser410Thr) variant**

Using NCBI Basic Local Alignment Search Tool (BLAST), the native and mutated sequences of Homo sapiens FAM20C gene were taken to study the effect of the variant, and also to predict the protein structure against PDB with default parameters [32]. The template PDBID: 5WRR was considered for modeling the protein structure. The native and mutant structure was modeled using a web server Swiss Model of Expasy [33,34]. Yet Another Scientific Artificial Reality Application (YASARA) was used to calculate the energy minimization of both the structures [35]. Stereochemistry and confirmation of the minimized structure were evaluated using VERIFY 3D [36, 37], ERRAT [38], PROCHECK [39] and Ramachandran plot through the Structural Analysis and Verification Server (SAVES) (<https://services.mbi.ucla.edu/SAVES/)>. The superimposition of the minimized structure of both native and mutant sequences was done using Discover studio, and the root mean sequence deviation (RMSD) was calculated. Online servers such as IStable [40] and I-Mutant Suite [41] with default parameters were used to check the protein stability.

The sequence identity for both the native and mutant of the *FAM20C* sequence with the crystal structure (Homo sapiens; 5WRR.PDB) was found to be 51% and 50% respectively. SAVES Meta server validated the quality of the predicted 3D protein models. Through VERIFY 3D, it was observed that 85.3% of the residues had an average 3D-1D score >0.2 for native structure and mutant structure, and hence it passed the protein model. An overall quality factor of 97.75 for native structure and 97.26 for mutant structure was indicated by ERRAT. Through Ramachandran plot, it was observed that 99.7% residues of the modeled native structure were in the allowed region and 0.3% residues were in the disallowed region; whereas, 100% residues of the mutant structure were in the allowed region. The RMSD of 0.3 A^o^ was observed between native and mutant structure, indicating changes in the loop regions of the superimposed structure. A decrease in stability was predicted by iStable with a conf. score of 0.605. Also, I-Mutant predicted a large decrease in protein stability due to the variant p.Ser410Thr with DDG values of -1.37 Kcal/mol (Figure-3).
