# Supplementary material for: A case of Raine syndrome presenting with facial dysmorphy and review of literature
Source: BMC Med Genet. 2018 May 11;19:76. doi: 10.1186/s12881-018-0593-x (PMC5948820; doi:10.1186/s12881-018-0593-x)
Supplement: Supplementary file 4 — Conservation of the FAM20C p.Ser410Thr residue in orthologs. Conservation of the variant in orthologs and Homo sapiens. (DOCX 15 kb) [file 12881_2018_593_MOESM4_ESM.docx]

**Additional file-4**

**Conservation of the FAM20C p.S410T residue in orthologs**

The protein sequence of *Homo sapiens* (NP_064608) was aligned along with other species using an online multiple sequence alignment program – Clastal Omega (<https://www.ebi.ac.uk/Tools/msa/clustalo/>). It is observed that the orthologs protein sequences of *FAM20C* gene are highly identical to *H. sapiens* *FAM20C* gene protein sequence (*M. musculus*: 86%*, C. canadensis*: 89%*, H. glaber*: 89%*, P. Anubis*: 95%*, P. abelii*: 98%*, p. troglodytes*: 98%*, O. garnettii*: 91%*, E.F. caballus*: 89%*, C. cristata*: 89%*, C.I. familiaris*: 89%*, and T. chinensis*: 90%). The residue Serine (Ser) is conserved in the orthologs (Figure-4A). The phylogenetic tree depicts the evolutionary conservation of *FAM20C* gene in many species (Figure-4B).
