# Supplementary material for: A case of Raine syndrome presenting with facial dysmorphy and review of literature
Source: BMC Med Genet. 2018 May 11;19:76. doi: 10.1186/s12881-018-0593-x (PMC5948820; doi:10.1186/s12881-018-0593-x)
Supplement: Supplementary file 5 — Population screening for c.1228T>A (p.Ser410Thr) variant by ARMS-PCR. The file provides details of normal healthy individuals studied for variant (c.1228T>A (p.Ser410Thr)) by ARMS-PCR. (DOCX 15 kb) [file 12881_2018_593_MOESM5_ESM.docx]

**Additional file-5**

**Population screening for c.1228T>A (p.Ser410Thr) variant by ARMS-PCR**

200 unrelated population (100 males and 100 females) were studied to demonstrate the pathogenicity of c.1228T>A (p.Ser410Thr) variant, using the Amplification-Refractory Mutation System (ARMS) PCR technique. ARMS-PCR is a very cost-effective technique and is apt for detecting point mutation because of its ability to discriminate among templates that differ by a single nucleotide residue. DNA samples of the population of concentration 100 ng/µl were amplified using Thermal Cycler-2720. A common forward primer (5'CAACAACATCTGCTTCTACG3'), a wild-type reverse primer (5'CTTGCGCTTGTGGTAGGA3') and a mutant reverse primer (5'CTTGCGCTTGTGGTAGGT3') were used. A total of 30 cycles comprising initial denaturation (94°C; 5 minutes), denaturation (94°C; 30 seconds), annealing of wild-type primer (58°C; 30 seconds), annealing of mutant primer (59°C; 30 seconds), elongation (72°C; 45 seconds) and final elongation (72°C; 5 minutes). The amplification of the PCR product was confirmed through 2.5% agarose gel electrophoresis.

From the 200 subjects screened for the above genotype, we could not find any carrier for the c.1228T>A (p.Ser410Thr) variant.
